# Supplementary material for: Quantifying small GTPase activation status using a novel fluorescence HPLC-based assay
Source: J Biol Chem. 2025 Apr 24;301(6):108545. doi: 10.1016/j.jbc.2025.108545 (PMC12151225; doi:10.1016/j.jbc.2025.108545)
Supplement: Supporting Information [file mmc1.pdf]

Supporting Information

Title:

Quantifying small GTPase activation status using a novel fluorescence HPLC-based assay

Authors:

Makoto Araki, Yukika Kasuya, Kaho Yoshimoto, Toshiaki Katada, Kenji Kontani

Included materials:

Figure S1-S2

Table S1

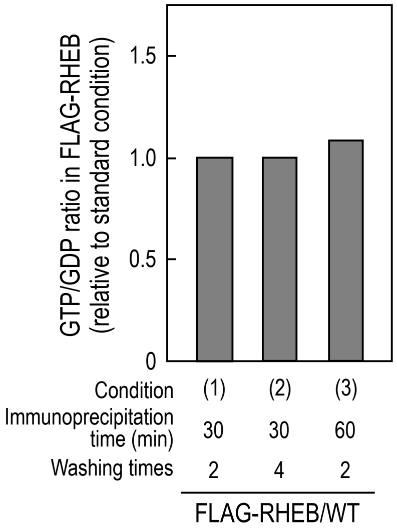

Figure S1. Effect of immunoprecipitation time and washing steps on GTP/GDP ratios in FLAG-RHEB.
HeLa cells expressing FLAG-RHEB were subjected to Fluor-HPLC analysis under three conditions: (1) standard conditions (30-minute immunoprecipitation with two washes), (2) increased washing steps (30-minute immunoprecipitation with four washes), and (3) extended immunoprecipitation time (1-hour incubation with two washes). The GTP/GDP ratios for each condition are shown from a representative experiment selected from multiple independent experiments.

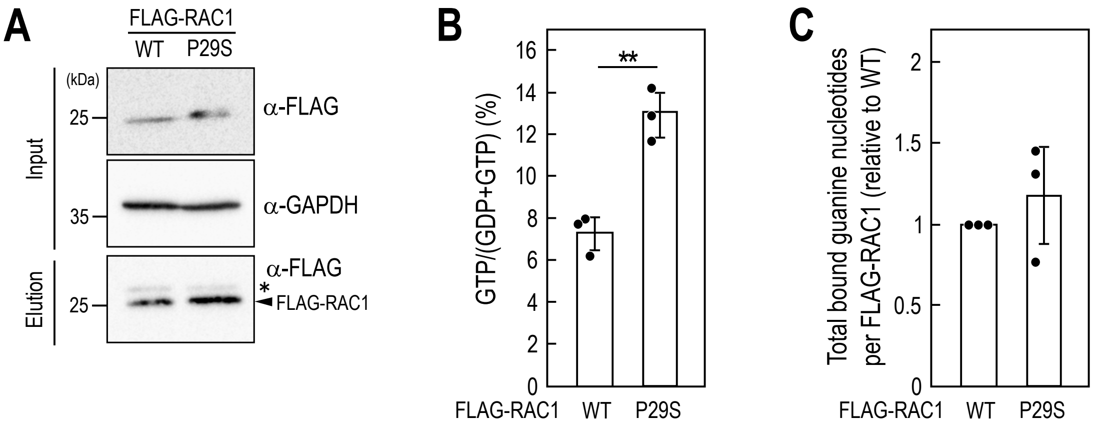

Figure S2. Analysis of the activation states of FLAG-RAC1 wild-type and the P29S mutant.
(*A*) The total cell lysates (*Input*) and anti-FLAG immunoprecipitates (*Elution*) prepared from HEK293T cells expressing FLAG-RAC1 wild-type (WT) or P29S mutant were subjected to western blot analysis using the indicated antibodies. The asterisk shows the light chain of anti-FLAG antibody. (*B*) Anti-FLAG immunoprecipitates from the cell lysates were subjected to Fluor-HPLC analysis, and the relative amounts of guanine nucleotides bound to FLAG-RAC1 were quantified based on the peak areas of GTP and GDP. Data represent the means ± SD from three independent experiments and indicate individual data points; ** P <0.01 by two-tailed unpaired Student's t-test. (*C*) Comparison of total bound guanine nucleotides in FLAG-RAC1 wild type and the P29S mutant. Anti-FLAG immunoprecipitates from HEK293T cells expressing FLAG-RAC1 (WT or P29S mutant) were analyzed by Fluor-HPLC to quantify total bound guanine nucleotides (GTP + GDP). The values were normalized to the levels of immunoprecipitated FLAG-RAC1 protein, as estimated by western blot analysis using an anti-FLAG antibody (band intensities were quantified by densitometric analysis with ImageJ software).

Table S1. Antibodies used in this study

Antibodies<br>[Working dilution]	Source	Identifier
RHEB<br>[1:2000]	Cell Signaling Technology	#13879, RRID:AB_2721022
S6K<br>[1:2000]	Cell Signaling Technology	#2708, RRID:AB_390722
Phospho-S6K (Thr389)<br>[1:2000]	Cell Signaling Technology	#9234, RRID:AB_2269803
TSC2<br>[1:2000]	Cell Signaling Technology	#4308, RRID:AB_10547134
Phospho-TSC2 (Thr1462)<br>[1:2000]	Cell Signaling Technology	#3617, RRID:AB_490956
ERK1/2<br>[1:2000]	Cell Signaling Technology	#4695, RRID:AB_390779
Phospho-ERK1/2 (Thr202/Thr204)<br>[1:2000]	Cell Signaling Technology	#4370, RRID:AB_2315112
DYKDDDDK (FLAG)<br>[1:2500]	Fujifilm Wako Pure Chemical	014-22383,<br>RRID:AB_10659717
GAPDH<br>[1:3000]	Fujifilm Wako Pure Chemical	016-25523,<br>RRID:AB_2814991
HRP-conjugated anti-rabbit IgG<br>[1:20000]	Jackson ImmunoResearch Labs	111-035-144,<br>RRID:AB_2307391
HRP-conjugated anti-mouse IgG<br>[1:20000]	Jackson ImmunoResearch Labs	115-035-146,<br>RRID:AB_2307392
